# Low-Temperature Direct Contact Membrane Distillation for the Treatment of Aqueous Solutions Containing Urea

**DOI:** 10.3390/membranes10080176

**Published:** 2020-08-03

**Authors:** Alessandra Criscuoli, Alfredo Capuano, Michele Andreucci, Enrico Drioli

**Affiliations:** 1 Institute on Membrane Technology (ITM-\CNR), via P. Bucci 17/C, 87036 Rende (CS), Italy; e.drioli@itm.cnr.it; 2 U.O.C. Nefrologia e Trapianto, A.O.U Federico II, 80131 Napoli, Italy; alfredo.capuano@unina.it; 3 Renal Unit–Department of Health Sciences of “Magna Graecia” University–Viale Europa, Campus Salvatore Venuta, 88100 Catanzaro, Italy; andreucci@unicz.it

**Keywords:** direct contact membrane distillation, urea, low temperature

## Abstract

Research activities on the application of direct contact membrane distillation (DCMD) for processing at low temperature (up to 50 °C) solutions containing urea were presented and discussed. Feeds were urine (also in mixture) and human plasma ultrafiltrate. Moreover, as a case study, the performance of membrane modules of different sizes and features was investigated for reaching the productivities needed in the treatment of the human plasma ultrafiltrate. In particular, two modules were equipped with the same type of capillaries, but differed in terms of membrane area, while the third module contained a different type of membranes and presented a membrane area in between those of the two previous modules. The three modules were compared, at a parity of operating temperatures and streams velocity, in terms of transmembrane flux, permeate production and size, underlining the directions to follow for a real implementation of the technique.

## 1. Introduction

**Figure 1:**
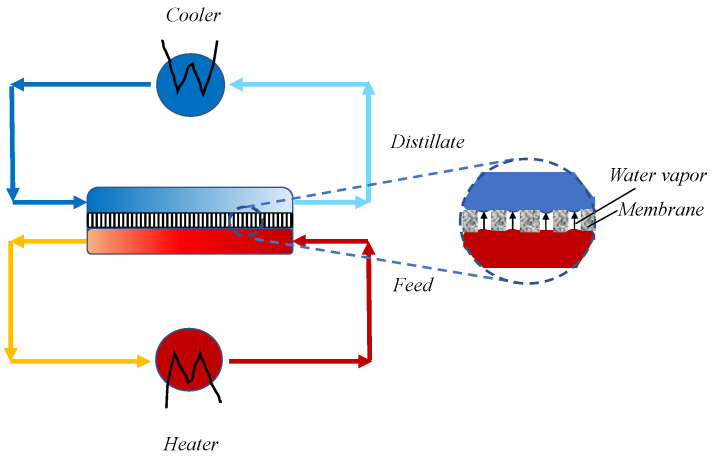
Direct contact membrane distillation (DCMD) process.

Direct contact membrane distillation (DCMD) is the most investigated configuration for various applications [1], because it is easy to implement and handle. In fact, the water vapor produced at the feed side is directly condensed at the distillate side, without requiring external condensers, as in vacuum membrane distillation (VMD) and Sweep Gas Membrane Distillation (SGMD). If compared with the air gap membrane distillation (AGMD) configuration, the DCMD operates with simpler membrane modules, in which only membranes are packed, and there is no need of condensing surfaces. In DCMD, one side of a microporous hydrophobic membrane is in direct contact with the feed to be distilled, while the other side contacts the distillate stream. The distillation occurs by applying a difference of temperature between the feed (hot stream) and the distillate (cold stream), resulting in a difference of vapor pressures across the membrane that promotes the transport of the water vapor from the feed to the distillate side through the dry membrane micropores (Figure 1).

During the distillation, the feed stream loses heat because of its evaporation, while the distillate stream warms up, due to the water vapor condensation. Therefore, at the exit of the membrane module, the streams are heated and cooled, respectively, to recycle them back to the membrane unit at the desired temperature values. The typical operating temperatures of the feed range between are 60 and 80 °C, in order to obtain a high difference of vapor pressures and then, high transmembrane fluxes, especially during concentration tests. However, for some applications, a lower temperature must be applied. In addition to the agrofood and pharmaceutical fields, lower operating temperatures are needed, for example, when treating biological fluids to avoid the denaturation and degradation of compounds. This is also the case of streams containing urea, for which most of the DCMD tests were carried out in the range of 40–50 °C. The purification of these streams is of interest for the wastewater treatment in space, for processing the wastewater coming from urea synthesis plants, as well as for the treatment of patients affected by chronic renal failure. In DCMD, it is possible to recover purified water, to re-used as distillate, and to produce a stream concentrated in urea, that can be further used in the production of fertilizers or in resin fabrication. The possibility to re-use the wastewater, including urine, in space is of high importance, because it avoids the need of external water supply and of wastewater storage/disposal [2]. Similarly, the recovery of water from the human plasma ultrafiltrate of patients undergoing extracorporeal blood purification techniques avoids the use of external water as dialysate and/or reinfusate fluid, strongly reducing the risk of inflammatory problems linked to the presence of chemical pollutants (even if in traces) [3]. The aim of this work was first to present and discuss the researches made in processing at low temperature (up to 50 °C) solutions containing urea by DCMD, specifically, urine (also in mixture) and human plasma ultrafiltrate. Then, as a case study, the results of experiments made to improve the productivity of the process for the treatment of the human plasma ultrafiltrate were reported. Tests were carried out on three commercial modules of different size (0.1, 0.35 and 0.83 m^2^) equipped with capillary polypropylene membranes (same membrane properties for the 0.1 m^2^ and 0.83 m^2^ modules). The efficiency of the modules was compared at a parity of operating temperatures and streams velocity, and the module with the best performance was identified.

## 2. Research Activities

The concept of DCMD was used for improving the performance of a combined forward osmosis (FO)–osmotic distillation (OD) unit for the treatment of metabolic wastewater in space [2]. Two systems were investigated: one where the MD membrane worked under a difference of temperature only (FO/MD), another where both a difference of temperature and concentration were applied (FO/membrane osmotic distillation (MOD). In the latter case, the permeate stream consisted in an osmotic agent (NaCl solution) rather than distilled water. In both systems, the MD membrane was laid on a semipermeable FO membrane. The synthetic wastewater was prepared including the main sources of wastewater on a spacecraft, like hygiene wastewater, humidity condensate and urine (urea, 5 g/L wastewater). The MD membrane was in flat configuration and made of polypropylene (PP; 0.22 μm of pore size, GE Osmonics, Minnetonka, MN). Tests on FO/MD were carried out on a solution of urea in deionized water and on a triply concentrated synthetic wastewater. The operating temperatures were kept at 25 and 21 °C for the feed and permeate, respectively. A constant flux of 0.8 L/m^2^ h was obtained for both feeds and a concentration factor of 9 was registered for the wastewater after about 70 h. Neither urea nor surfactant were found in the permeate, confirming the good rejections of the MD and FO membrane, respectively. The FO/MOD unit was tested at the same operating conditions of the FO/MD, but sending at the permeate side NaCl solutions (60–100 g/L). The flux was constant and around 0.9–1 L/m^2^h and also in this case a complete rejection of urea was obtained. In 15 days of test, the FO/MD system led to a 4–20 times higher flux than the FO/OD unit, while with the FO/MOD, a 8–25 times higher flux than the FO/OD unit was registered.

Always in the logic of wastewater recycling in space, DCMD tests were carried out by Cartinella et al. [4] on a mixture of humidity condensate and urine by using a commercial capillary module, MD020-CP-2N (Microdyn, Germany), of 0.1 m^2^ membrane area. The feed, at 40 °C, was sent inside the capillaries while the distilled water at 20 °C flowed countercurrently at the shell side. At a water recovery factor of 75%, the flux was about 1.5 L/m^2^h and the urea rejection was higher than 99.9%. Furthermore, the estrone and estradiol rejections were also analyzed and values higher than 99.5% were obtained.

The potential of DCMD coupled to FO was studied by Liu et al. [5] for the treatment of real human urine. The MD membrane was made of polytetrafluoroethylene (PTFE) and had a pore size of 0.45 μm (Jitian Company, Shangai, China). The DCMD unit used distilled water at the permeate side and had the scope of re-concentrating the draw solution (NaCl, 1–2.5 M), coming from the FO, and of producing the final distillate. In 8 h tests, the water transfer rate of the FO/MD system was around 3.39 L/m^2^ h at 40 °C and 1M NaCl as draw solution, and increased to 5.08 L/m^2^ h at 53 °C and 2.5 M NaCl. The overall rejection was nearly 100% for all contaminants (not only urea), due to the high rejection of MD for the non volatile species and of FO for the volatile ones. The concentrated urine can be used for nutrients recovery.

The integration of FO with DCMD was also investigated by Volpin et al. [6] who optimized the FO and DCMD operating parameters to reduce the nitrogen content in the produced distillate. In particular, for the DCMD unit, the effect of the feed temperature (from 40 to 60 °C) and membrane properties were analyzed, while keeping the cross-flow velocities (8.5 cm/s) and the temperature at the distillate side (20 °C) both constant. The membranes used were from Merk Millipore and had the same nominal pore size (0.22 μm), but differed in terms of material (polyvinylidene fluoride (PVDF) and PTFE), porosity and thickness (see Table 1). At all investigated temperatures, the PTFE membrane led to higher fluxes than the PVDF, due to the higher porosity and contact angle. Specifically, the transmembrane fluxes for the PTFE membrane and a 1.5 M NaCl feed varied from 6 L/m^2^ h to 12 L/m^2^ h at 40 and 50 °C, respectively, further increasing up to 16 L/m^2^ h at 60 °C. At the same operating conditions, the PVDF membrane led to fluxes of 4 L/m^2^h (40 °C), 7 L/m^2^ h (50 °C) and 11 L/m^2^ h (60 °C). The PTFE membrane was also able to give water fluxes higher than the NH_3_ flux, producing a high-quality distillate. 

Microporous hydrophobic composite membranes for water recovery from urine were prepared and tested by Khumalo et al. [7] who modified the PVDF/PTFE membranes with methyl functionalized silica nanoparticles (fMSNs). In particular, three membranes were prepared: M1-PVDF/0.3% fMSNs; M2- PVDF/3% PTFE/0.3% fMSNs; M3- PVDF/6% PTFE/0.3% fMSNs. DCMD tests were made on hydrolyzed human urine that was sent at one side of the membrane at 50 °C while deonised water circulated at the other side at 20 °C. The M3 membrane, with the highest contact angle and a less porous structure, performed better, showing higher rejections towards ammonia–nitrogen (99.1%), TOC (>98%), Na^+^ and K^+^ (>99%). A water recovery factor of 80% was obtained with fluxes similar to those reported in the literature at the same temperature difference. Nevertheless, the authors pointed out that fouling issues due to the possible deposition of urine compounds on the membrane surface must be taken into account during the treatment. 

The application of DCMD to purify the human plasma ultrafiltrate of patients affected by chronic renal failure was investigated by Capuano et al. [3], who used a commercial capillary module, MD020-CP-2N (Microdyn, Germany), of a 0.1 m^2^ membrane area. The experiments were made on two synthetic solutions containing urea and NaCl in different amounts (solution A: urea, 2 g/L and NaCl, 9 g/L; solution B: urea, 8 g/L and NaCl, 36 g/L), as well as on real human plasma ultrafiltrate (urea, 1.09 g/L; creatinine, 0.054 g/L; Na^+^, 3.27 g/L; K^+^, 0.129 g/L; Ca^2+^, 0.062 g/L). The feed was sent, at different flow rates (6–200 L/h) and temperatures (29–39 °C), inside the capillaries, while osmotic water recirculated counter-currently at the shell side. The highest transmembrane flux was 3 L/m^2^ h at 39 °C and 200 L/h of feed flow rate. In all tests, neither urea nor other species in the feed permeated through the membrane and the collected distillates were of high-quality. Furthermore, no fouling issues were registered, also during prolonged tests (lasted 4.5 h and 7 h) carried out on the real plasma ultrafiltrate. However, the permeate produced by the module (0.3 L/h) did not match the distillate needs for a clinical application of the technique, that ranged from 20 to 30 L/h. 

Table 1 summarizes the main results related to the research activities discussed. It is evident that only few studies were carried out on the application of low-temperature DCMD, and mainly at the lab-scale (membrane areas between 20 cm^2^ and 0.1 m^2^). Although the used membranes were mostly commercial, the modules were often lab-made and in flat configuration, with the exception of some tests made on a commercial capillary module [3,4]. The membranes were in PVDF, PTFE or PP, often with a typical pore size of 0.2 μm and a porosity between 70% and 85%, while their thickness varied from 125 to 450 μm. Nevertheless, all studies on the topic demonstrated an excellent rejection of DCMD for urea (up to 100%), confirming its purification and concentration capability. The use of the integrated FO/DCMD system resulted as beneficial for the final permeate quality, due to the FO rejection of volatiles, such as ammonia and nitrogeneous organic species, and surfactants, that preserved DCMD from wetting, combined with the high DCMD rejection of all non-volatiles, included urea.

## 3. Case Study: Improvement of the Permeate Production for the Treatment of the Human Plasma Ultrafiltrate by DCMD

Based on the results obtained in our previous work [3] which was presented in Section 2, through research activities, modules with higher membrane areas were investigated in order to increase the permeate production during the treatment of the plasma ultrafiltrate by DCMD. In particular, another module, produced by Microdyn (Wuppertal, Germany), the MD063CP2N, and a tailor made module (PF2000N), based on PF2000N design and containing a non-treated plasmaphan membrane (3M) provided by Gambro Dialysatoren GmbH (Hechingen, Germany), were tested at the same operating conditions. Hereinafter, the three modules will be identified as M1 (MD020CP2N), M2 (MD063CP2N) and M3 (PF2000N).

### 3.1. Materials and Methods

The M2 module was equipped with the same type of membranes of M1, in polypropylene, but was longer and contained a higher number of fiber. It has to be noticed that the data sheet of the Company reported a number of 200 fibers and a membrane area of 0.75 m^2^. However, the effective number of fibers was 220, so the membrane area used for the flux calculation was increased accordingly (0.83 m^2^) and also the free flow areas of the lumen and shell side were calculated considering the effective number of fibers. The M3 module contained a higher number of membranes (around 1500) made also of polypropylene, but with different properties and size. Its membrane area was in between those of the two previous modules (0.35 m^2^). The main properties of the membranes and modules are reported in Table 2; Table 3, respectively.

Both microdyn modules have 40 °C as the maximum operating temperature, while the M3 module is usually operated at around 37 °C. Therefore, the comparison experiments were carried out at around 37 °C of feed temperature. The permeate temperature was around 22 °C. The maximum operating feed and permeate flow rates that could be used with the M3 module without increasing the relative pressure values were 170 L/h and 250 L/h, respectively. The corresponding velocities were 0.35 m/s for the feed stream and 0.16 m/s for the permeate side. The operating feed and permeate flow rates to be used in the two Microdyn modules were then calculated, considering these velocity values. It has to be noticed that, with the above feed velocities, the three modules worked in laminar regime, with the Reynolds values being equal to 154 and 840 for the M3 and the two microdyn modules, respectively.

### 3.2. Mass Transfer 

In DCMD, the water vapor mass flux is a function of the membrane properties, as well as of the difference of water vapor pressure across the membrane (driving force) and can be described as

(1) $$J=K\times \left(P_{\mathrm{fm}}-P_{\mathrm{dm}}\right)$$

where J (kg/m^2^s) is the transmembrane flux, K (kg/m^2^ s Pa) is the membrane distillation coefficient, P_fm_ (Pa) is the feed vapor pressure at the membrane surface-hot side, and P_dm_ (Pa) is the distillate vapor pressure at the membrane surface-cold side. The membrane distillation coefficient includes the membrane properties. They are usually assembled in two ratios based on two main mechanisms that can occur separately or in combination, depending on the membrane pore size and operating conditions: Knudsen and molecular diffusion. The type of mechanism occurring in the membrane is usually identified by calculating the Knudsen coefficient as the ratio between the mean free path of molecules and the mean pore size of the membrane. When the Knudsen coefficient is higher than 1, the Knudsen transport dominates, while for Knudsen coefficient values lower than 0.01, the molecular diffusion takes place. For Knudsen coefficient values between 0.01 and 1, both mechanisms can occur (transition region) [8]. The mean free path of water vapor can be calculated by [8]

(2) $$l=\frac{k_{B}T}{\pi {\left(\frac{\sigma_{w}+\sigma_{a}}{2}\right)}^{2}P_{\mathrm{pore}}\sqrt{1+\left(\frac{M_{w}}{M_{a}}\right)}}$$

where k_B_ is the Boltzman constant (J/K), T is the mean temperature in the pores (K), σ_w_ is the collision diameters of water vapor (m), σ_a_ is the collision diameters of air (m), P_pore_ is the air pressure in the pores (kPa), M_w_ is the water molecular weight (g/mol), and M_a_ is the air molecular weight (g/mol).

### 3.3. Specific Thermal Energy Consumption (STEC) and Productivity/Size (PS) Ratio

An important aspect in membrane distillation is the thermal energy consumption linked to the heat of the feed stream [9]. In this respect, the STEC is often used, calculated as the energy to supply to the feed recirculating inside the module, divided by the permeate production:

(3) $$\mathrm{STEC}=\frac{Q_{f}\times c_{p}\times \left(T_{\mathrm{fin}}-T_{\mathrm{fout}}\right)}{Q_{p}}$$

where Q_f_ is the feed flow rate (kg/h), c_p_ is the specific heat of the feed (kJ/kg K), T_fin_ is the feed temperature at the module inlet (K), T_fout_ is the feed temperature at the module outlet (K), and Q_p_ the permeate flow rate (L/h).

Another important parameter is the size of the membrane distillation plant needed for obtaining a certain productivity. In the process intensification strategy, future plants should ensure high productivities and high compactness. In this logic, a metric was defined to compare the plants in terms of productivity and size. In particular, the productivity/size ratio (PS) metric, compares the ratio of the productivity and size of two plants [10]:

(4) PS ProductivitySize| plant1ProductivitySize| plant2

When the PS is higher than 1, plant 1 must be preferred, otherwise, plant 2 must be chosen.

### 3.4. Experimental Set-Up and Procedure

DCMD tests on the different modules were carried out on the same lab set-up.

As already mentioned, the case study was based on the results obtained in our previous work [3], where both artificial solutions containing urea (0.2 wt % and 0.8 wt %) and real ultrafiltrate samples (urea content: 0.1 wt %) were treated by the M1 module. These experiments were carried out in the feed flow rate range of 6–200 L/h, corresponding to Reynolds values of 39 and 1292, respectively. In that study, the urea was completely rejected and no fouling issues were observed, also in prolonged tests on real ultrafiltrate samples. In the case study of the present work, the M2 module was equipped with the same fibers of the M1, so the same behaviour with solutions containing urea is expected. The M3 module was also equipped with fibers made of polypropylene, thus, the interactions between the urea and the membrane material can be considered the same as for the other two modules. Furthermore, the M3 module was operated at the same feed velocities as the M1 and M2 modules and the Reynolds values involved (154 for the M3 and 840 for the Microdyn modules) fell in the range investigated in our previous work. Finally, it has to be noticed that the feed of interest is quite diluted (the human plasma ultrafiltrate contains about 0.1 wt % of urea, that is highly soluble in water). On the basis of the above considerations, the experiments to investigate the possibility of increasing the permeate productivity were carried out using distilled water as both feed and permeate stream.

**Figure 2:**
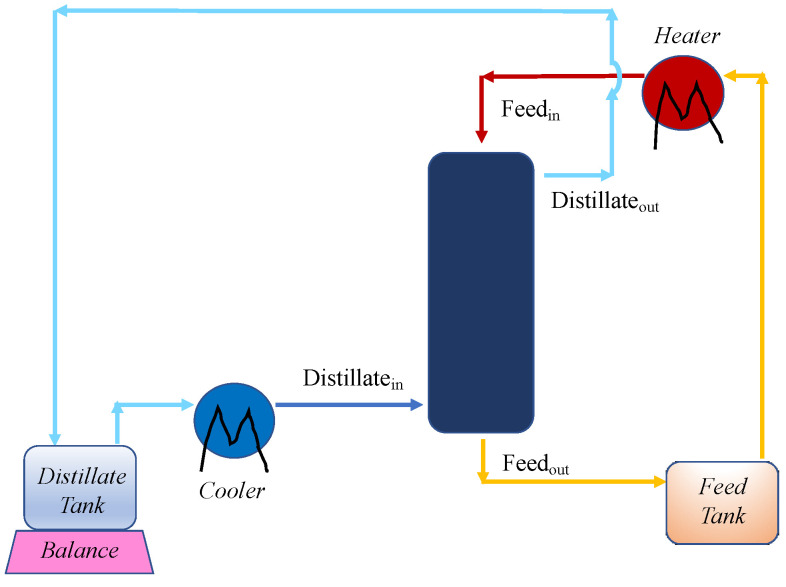
How the streams were fed to the M2 module in the lab set-up.

The two streams were re-circulated in counter-current flow mode to the module (the feed in the lumen and the permeate in the shell), after their heating and cooling, respectively. In the set-up, the module inlet and outlet temperatures were measured for both hot and cold lines and the operating pressures were monitored by two manometers, located at the two entrances of the module. All tests were carried out at atmospheric pressure. The permeate flux was calculated by registering the weight change of the distillate tank, located on an electronic balance, and by dividing the accumulated mass by the membrane area and the operating time. The experiments lasted one hour and no significant changes in the flux values were registered in time. The M1 and M3 modules were used in horizontal position, for an immediate fixing with the existing tubes of the lab set-up, whilst the M2 module, having a bigger size, was mounted in vertical and fed from the bottom with the permeate stream, to ensure that all the shell side was wetted and that no channeling occurred (Figure 2). It has to be mentioned that both the M1 and M3 modules can also be used in vertical position, as M2.

### 3.5. Results and Discussion

**Figure 3:**
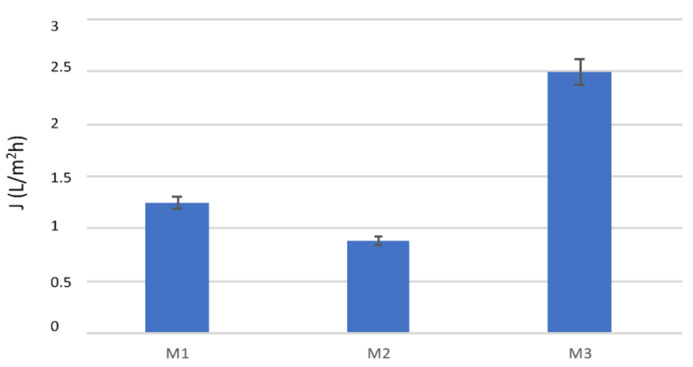
Transmembrane flux obtained with the three modules. v_fin_, 0.35 m/s; v_din_, 0.16 m/s; T_fin_, 37 °C; T_din_, 22 °C.

**Figure 4:**
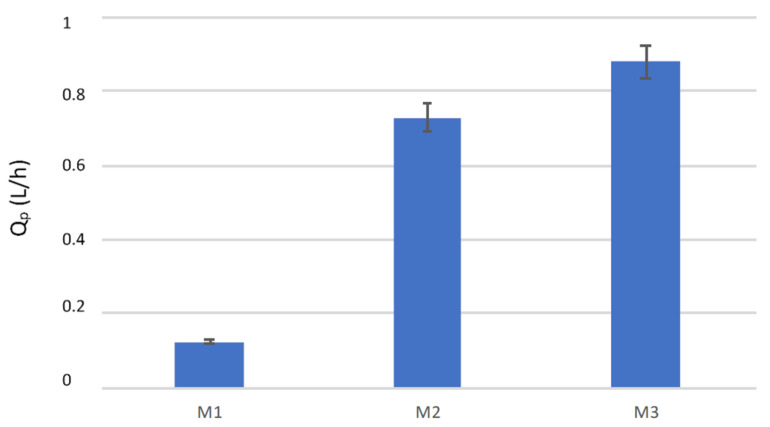
Permeate produced with the three modules. v_fin_, 0.35 m/s; v_din_, 0.16 m/s; T_fin_, 37 °C; T_din_, 22 °C.

The transmembrane flux obtained with the three modules operating at the same conditions is shown in Figure 3. A comparison of the modules in terms of productivity (Qp) is reported in Figure 4.

The highest flux was achieved with the M3 module, followed by the M1, and the M2 module leading to the lowest value. Since the two Microdyn modules were equipped with the same type of membrane, a similar flux was expected if working at the same inlet conditions. However, a 29% lower flux was registered. This result can be due to the longer fibers of the M2 module (the M2 length was 75 cm vs. 50 cm of the M1), as well as to its not uniform packing of capillaries. When working with longer membranes, the feed and permeate temperatures along the module decrease and increase, respectively, more significanlty than with shorter membranes, and therefore, a lower driving force is available for the vapor transport. Furthermore, the worse result of the M2 module could also be attributed to a bad flow distribution of the permeate stream at the shell side, because of the bad packing of the capillaries that caused a non-uniform flow along the module and, possibly, a reduction of the effective membrane area available for the permeation where adjacent capillaries touch each other. If part of the outer membrane surface is not in contact with the permeate stream or if the permeate stationates/slowly flows inside empty spaces present among membranes, the vapor coming from the feed side is not efficiently condensed and removed, so that the resistance to the vapor transport is increased and heat accumulates at the shell side, with a consequent reduction in the driving force available. To support this hypothesis, the distribution of the fibers inside the two modules was inspected.

**Figure 5:**
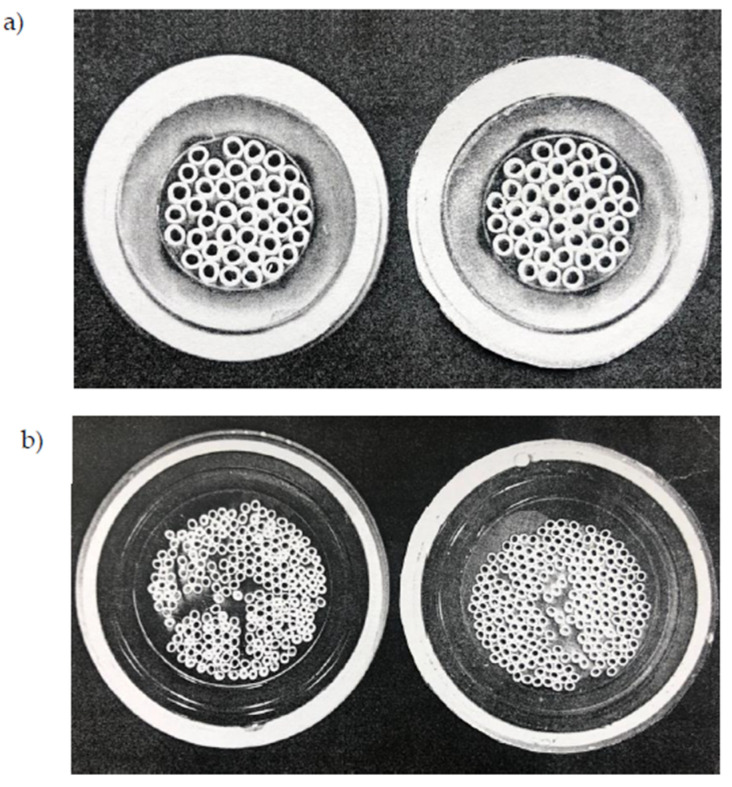
Picture of the (**a**) the M1 and (**b**) the M2 modules.

In Figure 5, the pictures of the two ends of each module are shown. It is evident that the capillaries were well and evenly packed in the smaller module, whilst in the bigger module, they were not uniformly distributed. In fact, some of them were very close and some others were quite far, leaving empty spaces. It is expected that the same happens inside the module, where it is difficult to ensure a fixed distance among capillaries.

**Figure 6:**
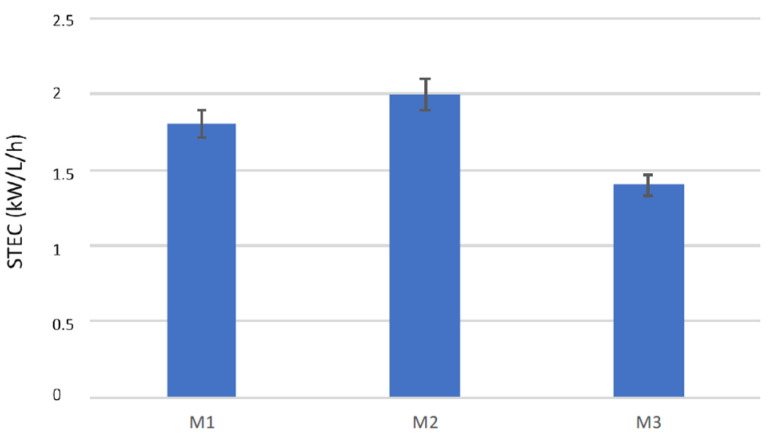
Specific thermal energy consumption (STEC) values for the three modules.

Therefore, with the two Microdyn modules, it was not possible to operate in a modular way: by working with a 8.3 higher membrane area, the productivity was increased by a factor of 5.9. The mean free path of the water vapor at 37 °C calculated by equation (2) was 0.1075 μm and the Knudsen coefficient varied between 0.36 and 0.54 for the M3 and the Microdyn membranes, respectively, indicating in both cases a transition region for the mass transport. The highest flux and productivity of the M3 module can be attributed to the different membrane properties, that presented a higher pore size and lower thickness than the capillary membranes used in the Microdyn modules. It has to be pointed out that the M3 module is also the shortest one, which is a favorable feature to reduce the feed temperature decay along the fibers. The three modules were also compared in terms of STEC. In particular, Figure 6 shows the STEC for the three modules operating at the same conditions. The M3 module led to the lowest STEC, due to the highest permeate production, while the two Microdyn modules had similar STEC values, although that of the M1 module was slightly lower. The STEC data are an indication of the energy efficiency of the modules, as they give an information of the thermal energy needed for a certain permeate production. Therefore, the lowest is the STEC, the highest is the energy efficiency of the unit. It has to be noticed that in lab-scale tests the STEC values are usually higher than those obtained with bigger membrane modules, due to the low membrane areas involved and the related low permeate produced. Nevertheless, in this work, due to the higher transmembrane fluxes, the M3 module (0.35 m^2^) was able to produce more permeate than the M2 module (0.83 m^2^), leading to their best performance. This result points out the importance of membrane properties and module features for improving the efficiency of membrane distillation units.

**Figure 7:**
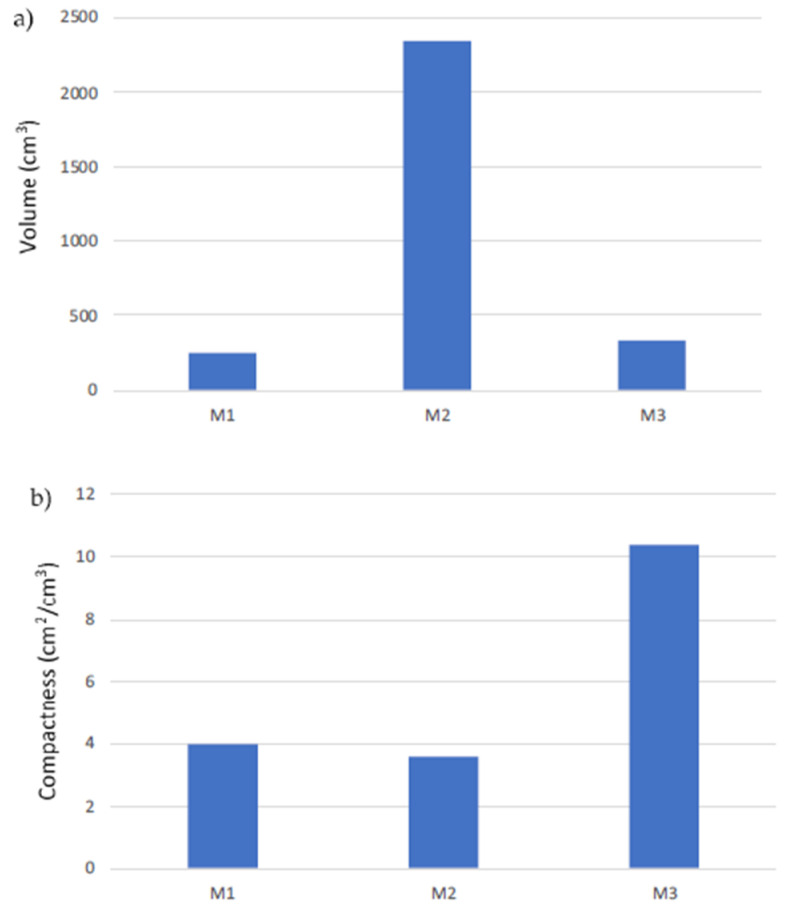
(**a**) Size (volume) and (**b**) compactness of the three modules.

The size of the membrane distillation modules is also important, in the logic of the reduction of the land use and of the minimization of the occupied volume in space applications (process intensification strategy). Figure 7a compares the modules in terms of volume, showing that the two Microdyn modules had the highest (M1) and lowest (M2) volumes, with the M3 volume closer to the M1 one. In Figure 7b, the compactness (calculated as the membrane area divided by the volume) is reported for the three modules. The M2 was the less compact, followed by the M1 and finally by the M3 module, that was the most compact one. This result confirms the benefit of using smaller capillaries that allow to achieve high membrane area values in lower volumes.

**Figure 8:**
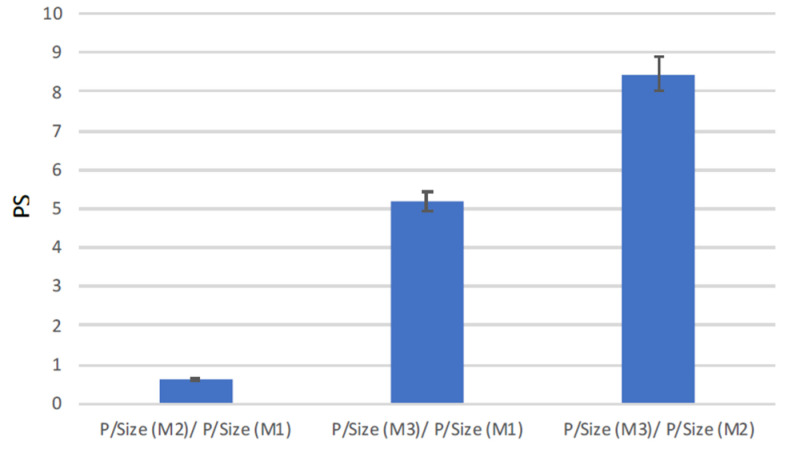
Modules’ comparison in terms of the productivity/size ratio (PS).

Based on the obtained data, the three modules were finally compared in terms of PS, see Figure 8. The PS was calculated according to Equation (4), with the permeate flow rate Qp as the productivity and the volume of the module as size.

With respect to the M1 module, the M2 presented a lower productivity per size (PS < 1), while the M3 module showed the best performance, leading to ratios much higher than 1 if compared with both Microdyn modules.

## 4. Conclusions

The potential of DCMD for treating at low temperature solutions containing urea was explored in few studies and therefore, the state of the art on this topic is still limited. Nevertheless, the researches made in the field confirmed the high rejections for urea of DCMD, that can be used to produce purified water together with a concentrate source for urea/nutrients recovery. This result is interesting for applying/improving the recycle and recovery concept in space engineering, in medical treatments, as well as in urea wastewater treatment plants. 

Concerning the case study on the use of DCMD for recovery water from the human plasma ultrafiltrate, the 0.35 m^2^ (M3) module led to the highest permeate production (0.88 L/h) and to the lowest STEC (1.4 kW/L/h), resulting also to be the most compact and with the lowest size. However, the productivity target is still far and therefore, there is the need of developing capillary membranes and modules “ad hoc”, in order to obtain the desired distillate production. 

From the carried out investigation, the difficulty in scaling-up capillary membrane modules, to ensure modularity, is evident. Nevertheless, some inputs for addressing membrane and module developments can be derived: specific attention should be made to the optimal size of the membranes, because small diameters allow a high compactness and low size of the module, but could limit the operating flow rates, due to the pressure drops, with a consequent increase in polarization phenomena and flux reduction. The permeate production can be also enhanced by using membranes with higher porosity and pore size and lower thickness, taking care of the wetting risk. Referring to the module development, shorter modules should be preferred, in order to reduce the feed temperature decay and to avoid capillary deformation. A fundamental step is to also ensure an uniform packing of the membranes inside the module, so that to have a constant cross section for the fluid flow and to effectively utilize all the membrane area for the distillation process. 

## Figures and Tables

**Table 1:** Main results of the research activities on the treatment of solutions containing urea by low-temperature DCMD.

Urea Source	Module Geometry	Membrane Properties	Operating Conditions	Main Results	Refs.
Feed 1: Solution of urea; Feed 2: Triply concentrated synthetic wastewater	Flat	PP; d_p_: 0.22 μm; ε: 70%; δ: 150 μm; A_m_: 139 cm^2^	T_f_: 25 °C; T_d_: 21 °C; v_f_: 0.1 m/s	FO/MD: J: 0.8 L/m^2^h; R_urea_: 100%; Conc. factor (70 h): 9 FO/MOD: J: 0.9–1 L/m^2^h; R_urea_: 100%	[2]
Synthetic mixture of humidity condensate and urine	Capillary	PP; d_p_: 0.2 μm; ε: 70%; δ: 450 μm; A_m_: 0.1 m^2^	T_f_: 40 °C; T_d_: 20 °C; Q_f_: 90 L/h	Water recovery factor: 75%; J: 1.5 L/m^2^h; R_urea>_99.9%	[4]
Real human urine	Flat	PTFE; d_p_: 0.45 μm; δ: 180 μm; A_m_: 29.5 cm^2^	T_f_: 40–55 °C; T_d_: 25 °C; Q_f_: 12 L/h	FO/MD (40 °C; 1 M NaCl): J: 3.39 L/m^2^h; R_urea_: 100%	[5]
Synthetic urine	Flat	PVDF; d_p_: 0.22 μm; ε: 75%; δ: 125 μm; PTFE; d_p_: 0.22 μm; ε: 85%; 150 μm; A_m_: 20 cm^2^	T_f_: 40–60 °C; T_d_: 20 °C; v_f_: 0.085 m/s	FO/MD (40 °C; 1.5 M NaCl): J_PVDF_: 4 L/m^2^h; J_PTFE_: 6 L/m^2^h	[6]
Hydrolyzed real human urine	Flat	PVDF/6%PTFE/0.3%fMSNs; Contact angle: 115.5°; A_m_: 125 cm^2^	T_f_: 50 °C; T_d_: 20 °C; Q_f_: 35 L/h	Water recovery factor: 80%; R_ammonia-nitrogen_: 99.1%	[7]
Feed 1: synthetic solution of urea and NaCl Feed 2: real human plasma ultrafiltrate	Capillary	PP; d_p_: 0.2 μm; ε: 70%; δ: 450 μm; A_m_: 0.1 m^2^	T_f_: 29-39 °C; T_d_: 13–20 °C; Q_f_: 6–200 L/h	J (39 °C; 200 L/h): 3 L/m^2^h; R_urea_: 100%	[3]

d_p_: mean membrane pore size; ε: membrane porosity; δ: membrane thickness; A_m_: membrane area; T_f_: feed temperature; T_d_: distillate temperature; v_f_: feed velocity; Q_f_: feed flow rate; J: transmembrane flux; R: rejection; PP: polypropylene; PTFE: polytetrafluoroethylene; PVDF: polyvinylidenefluoride; FO/MD: forward osmosis/membrane distillation; MOD: membrane osmotic distillation.

**Table 2:** Main properties of the membranes.

Module	Membrane Material	Pore Size (μm)	Porosity (%)	Thickness (mm)	Inner Diameter (mm)	Outer Diameter (mm)
M1/ M2	Polypropylene	0.2	70	0.45	1.8	2.7
M3	Polypropylene	0.3	70	0.15	0.33	0.63

**Table 3:** Main properties of the modules.

Module	Number of Fibers	Membrane Area (m^2^)	T_max_ (°C)	Size (cm × cm)
M1	40	0.1	40	50 × 2.5
M2	220	0.83	40	75 × 6.3
M3	1555	0.35	50	25 × 4.14
